# Functional, Non-Clonal IgM^a^-Restricted B Cell Receptor Interactions with the HIV-1 Envelope gp41 Membrane Proximal External Region

**DOI:** 10.1371/journal.pone.0007215

**Published:** 2009-10-06

**Authors:** Laurent Verkoczy, M. Anthony Moody, T. Matt Holl, Hilary Bouton-Verville, Richard M. Scearce, Jennifer Hutchinson, S. Munir Alam, Garnett Kelsoe, Barton F. Haynes

**Affiliations:** 1 Human Vaccine Institute, Duke University Medical Center, Durham, North Carolina, United States of America; 2 Department of Immunology, Duke University Medical Center, Durham, North Carolina, United States of America; New York University School of Medicine, United States of America

## Abstract

The membrane proximal external region (MPER) of HIV-1 gp41 has several features that make it an attractive antibody-based vaccine target, but eliciting an effective gp41 MPER-specific protective antibody response remains elusive. One fundamental issue is whether the failure to make gp41 MPER-specific broadly neutralizing antibodies like 2F5 and 4E10 is due to structural constraints with the gp41 MPER, or alternatively, if gp41 MPER epitope-specific B cells are lost to immunological tolerance. An equally important question is how B cells interact with, and respond to, the gp41 MPER epitope, including whether they engage this epitope in a non-canonical manner i.e., by non-paratopic recognition via B cell receptors (BCR). To begin understanding how B cells engage the gp41 MPER, we characterized B cell-gp41 MPER interactions in BALB/c and C57BL/6 mice. Surprisingly, we found that a significant (∼7%) fraction of splenic B cells from BALB/c, but not C57BL/6 mice, bound the gp41 MPER via their BCRs. This strain-specific binding was concentrated in IgM^hi^ subsets, including marginal zone and peritoneal B1 B cells, and correlated with enriched fractions (∼15%) of gp41 MPER-specific IgM secreted by *in vitro*-activated splenic B cells. Analysis of Igh^a^ (BALB/c) and Igh^b^ (C57BL/6) congenic mice demonstrated that gp41 MPER binding was controlled by determinants of the Igh^a^ locus. Mapping of MPER gp41 interactions with IgM^a^ identified MPER residues distinct from those to which mAb 2F5 binds and demonstrated the requirement of Fc C_H_ regions. Importantly, gp41 MPER ligation produced detectable BCR-proximal signaling events, suggesting that interactions between gp41 MPER and IgM^a^ determinants may elicit partial B cell activation. These data suggest that low avidity, non-paratopic interactions between the gp41 MPER and membrane Ig on naïve B cells may interfere with or divert bnAb responses.

## Introduction

A major roadblock in generating an effective HIV-1 vaccine has been the inability to elicit broadly neutralizing antibodies (bnAbs), capable of neutralizing a diverse variety of HIV-1 primary isolates, either during acute HIV-1 infections, or after immunization with various HIV-1 envelope (Env) immunogens [1]–[4]. Explanations for the poor immunogenicity of HIV-1 Env include the emergence of a glycan shield covering large portions of gp120 [5]–[8], conformational shifting [5], [9], and the high mutation rates of HIV-1 Env structural genes [7], [10]. Significant advances in studying the capacity of humans to make protective Ab include the isolation of rare human bnAbs isolated from HIV-1 infected patients, such as 2F5, 4E10, 2G12, 1b12 and Z13, and the characterization of their interactions with various regions of the HIV Env [11]. Passive infusion of mixtures of 2F5, 4E10, 1b12 or 2G12 into rhesus monkeys protects against SHIV infection, providing hope that if these antibodies could be induced in man, a preventive HIV-1 vaccine might be possible [12], [13].

Two of these rare bnAbs, 2F5 and 4E10, interact with a particularly attractive target for a B cell-based HIV-1 vaccine, the HIV-1 gp41 membrane proximal external region (MPER), in the neighboring linear, neutralizing core epitopes ELDKWA and NWFDIT, respectively [2], [14]–[22]. Although immunization with Env subunit immunogens containing gp41 MPER core epitopes can elicit non-neutralizing gp41 MPER antibodies, they have not elicited bnAbs in any species [17], [23]–[27]. One possibility why gp41 MPER-containing immunogens fail to elicit bnAbs is that they are not in native conformations. In particular, there is evidence that the physiological form of the gp41 MPER to which 2F5 binds, the “prehairpin intermediate”, is a unique structure that is only transiently expressed upon engagement of the CD4 receptor and chemokine co-receptors [11], [28]–[30], implying that the repertoire of gp41 MPER bnAb-producing B cells is present, but the gp41 MPER pre-hairpin intermediate form is not available for a sufficient period of time to engage B cells.

The bnAb 2F5 and 4E10 have long hydrophobic CDR3 regions [16], [18] and exhibit polyreactivity to lipids and other autoantigens [31]–[33]. Thus, a second possibility for poor MPER immunogenicity is that gp41 MPER-specific bnAbs cannot be elicited because the B cells from which they originate are subjected to peripheral or central B cell tolerance mechanisms [34]. Such mechanisms could include deletion, anergy, or receptor editing and could be due to gp41 MPER cross-reactivity with as yet unidentified self or environmental/commensural antigens and/or to the viral lipid membrane [35], [36].

While non-neutralizing gp41 MPER Ab responses have been studied, virtually nothing is known about the B cell populations that interact with and/or respond to the broadly neutralizing epitopes of HIV-1 Env. One key question is whether MPER epitope-specific B cells are lost to tolerance at some point during B-cell ontogeny [35], [37]. Another fundamental question is whether B-cell interactions with gp41 MPER occur solely via paratopic recognition by B cell receptors (BCR). For example, B cells can interact with other parts of the HIV-1 Env through BCR-independent mechanisms, such as binding gp120 outside conventional antigen-binding regions in Ig [37] or via mannose C type lectin receptors [38].

The goal of this study was to define B cell interactions with the gp41 MPER epitope of one of the most potently and broadly neutralizing antibodies known, the rarely-made mAb 2F5, and to compare it with B cell interactions occurring with other HIV-1 epitopes. We chose to study murine B cells, since failure to induce bnAbs to the gp41 MPER is not species-specific, and 2F5 cross-reacts with human and murine self-antigens ([32] and G. Kelsoe, unpublished results). Moreover, studies of B cell genetics and ontogeny can be simplified in the mouse, where congenic strains and developmental B-cell subsets are well-defined. In this study, we have used tetramerized HIV-1 peptide epitopes [39] to identify a novel type of interaction between BALB/c BCRs and the gp41 MPER. Characterization of this interaction reveals that it is non-paratopic and instead mediated by Cμ determinants expressed on IgM^a^ but not IgM^b^ polypeptides. In addition, whereas this frequent, non-paratopic binding of gp41 MPER represents a low avidity interaction, it is sufficient to partially activate splenic B cells *in vitro*. Finally, we demonstrate preferential gp41 MPER-specific IgM^+^ Ab production in polyclonally-activated BALB/c gp41 MPER-binding splenic B cells and in naïve BALB/c serum, suggesting that natural non-neutralizing anti-gp41 MPER-specific Abs are spontaneously produced by MZ and B1 IgM^a^ B cells.

## Results

### Generation and validation of gp41 MPER B cell tetramers

The 2F5 mAb, like all other HIV-1 bnAbs, is not normally made during the course of natural HIV-1 infection, nor can it be elicited in any species immunized by current HIV-1 immunogens. However, non-neutralizing Abs specific for the gp41 MPER 2F5 epitope can readily be elicited by immunization in various species, including mice ([23], [40] and Haynes and Verkoczy, unpublished results). To begin understanding what may be inherently different about 2F5 (or 2F5-like) broadly neutralizing and non-neutralizing Ab responses, we sought to characterize what B cell populations, if any, specifically bound the gp41 MPER 2F5 epitope, and if such interactions differed from those with 2F5 itself. To do this, we generated APC-labeled tetramer reagents containing the gp41 MPER 2F5 epitope to allow for convenient and sensitive detection of B cells by flow cytometry, by analogy to studies using tetramers for identifying antigen-specific T cells [41]. We reasoned that tetramerization of the gp41 MPER 2F5 epitope would i) more physiologically mimic the multimeric nature of BCR interactions with antigens, and ii) allow us to identify B cells with a larger range of affinities for the 2F5 gp41 MPER epitope because of the increased avidity conferred by its multimerization.

For these studies, tetramers specific for the 2F5 epitope of gp41 MPER (herein referred to simply as gp41 MPER) were made from the peptide QQEKNEQELLELDKWASLWN; (corresponding to residues 659–678 of the HIV-1 envelope). This peptide was chosen because in addition to containing the original ELDKWA core neutralization motif of 2F5, it contains additional residues flanking this region which have been shown by several mapping/affinity measurement studies to confer optimal binding of 2F5 to gp41 MPER [14], [40], [42], [43] and thus represents the ideal, nominal 2F5 epitope. The specificity and quality controls to validate gp41 MPER-specific B-cell tetramers, as well as tetramer reagents to other well-characterized HIV-1 Env epitopes, are summarized in Fig. S1.

### The gp41 MPER epitope interacts with large fractions of naïve splenic B cells in a strain-specific manner

**Figure 1:**
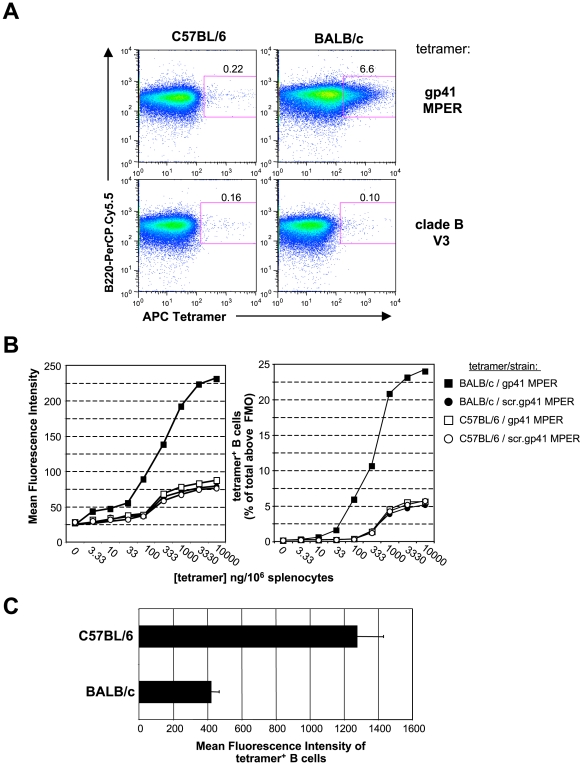
Strain-specific gp41 MPER interactions with B cells. A) Representative histograms showing surface staining of 10^6^ splenocytes from unimmunized C57BL/6 and BALB/c mice with 100 ng of APC-labeled gp41 MPER or clade B V3 epitope-specific tetramers. Shown are singlet, live, total B cell (CD19^+^)-gated splenocytes. Numbers indicate percentages of tetramer-reactive B cells above background staining without tetramer. B) Tetramer binding analysis of splenic B cell populations from unimmunized C57BL/6 and BALB/c mice using serial dilutions of APC-labeled gp41 MPER or scrambled gp41 MPER epitope-specific tetramers to stain 10^6^ splenocytes. APC MFIs or percentages of tetramer-reactive cells within singlet, live, CD19^+^ gates were measured and graphically represented. Data shown are representative of several experiments. C) Comparison of binding avidity of C57BL/6 and BALB/c total splenic B cells to the gp41 MPER, as measured by APC MFIs of tetramer-reactive B cells. gp41 MPER tetramers (100 ng/10^6^ cells) were used to surface-stain splenocytes and tetramer-reactive B cell MFIs (above staining with no tetramer) within the singlet, live, CD19^+^ gate was calculated and graphically represented. Data shown are taken from a representative experiment (mean±s.d of five mice).

We first identified if and how splenic B-cell populations in naïve BALB/c and C57BL/6 mice bound the APC-labeled gp41 MPER tetramer by flow cytometry. We used mouse B cells to study since the inability to elicit 2F5 or “2F5-like” bnAbs, as stated above, is not a species-specific issue, yet B cell genetics and development are best defined in mice, and thus we reasoned are best-suited starting models to study this fundamental question. Strikingly, a large fraction (∼7%) of naïve splenic BALB/c B cells bound the gp41 MPER tetramer (Fig. 1A, upper right panel). In contrast, frequencies of MPER binding by naïve splenic C57BL/6 B cells was only 0.1–0.3% (Fig. 1A, upper left panel). Binding frequencies of a control tetramer bearing an epitope from a conserved portion of the gp120 V3 loop were low in both strains and comparable to observed for MPER-binding by C57BL/6 splenic B cells. (Fig. 1A, bottom panels).

We explored the nature of this strain-specific binding of gp41 MPER by labeling BALB/c and C57BL/6 splenic B cells with gp41 MPER tetramer or tetramers bearing scrambled MPER peptides over a wide concentration range. Elevated gp41 MPER binding to BALB/c cells, relative to C57BL/6 B cells, was observed at all concentrations of tetramers carrying MPER peptide, as measured both by mean fluorescence intensity (MFI, Fig. 1B, left panel) and the fraction of labeled B cells (Fig. 1B, right panel). This MPER-specific binding was substantially elevated relative to the scrambled peptide controls, ruling out binding determined solely by charge or other general properties. Importantly, saturating levels of gp41 MPER binding was attained at 10 µg/10^6^ splenocytes, when ∼25% of all B cells were labeled, the highest gp41 MPER/scrambled gp41 MPER binding ratio was achieved at the sub-saturating concentration of 100 ng/10^6^ splenocytes. For this reason, we employed this concentration of tetramer reagent for all subsequent studies.

### gp41 MPER-reactive BALB/c B cells are concentrated in marginal zone and B1 B cell subsets

**Figure 2:**
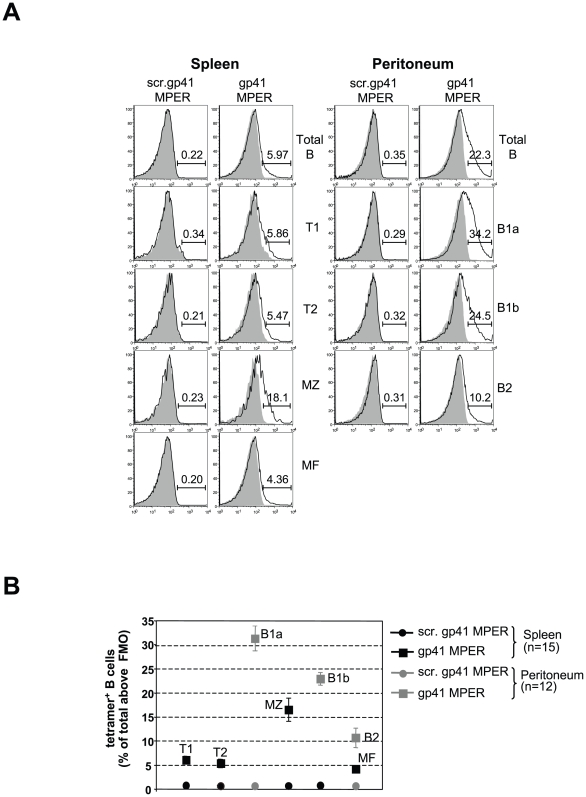
Relative frequencies of gp41 MPER-reactive cells in B cell subsets. A) Representative surface staining with gp41 MPER tetramers (100 ng/10^6^ cells) in splenic and peritoneal B cell subsets from naïve BALB/c mice. For each subset, shown are open histograms indicating either the APC-labeled scrambled gp41 MPER tetramer (left panels), or the gp41 MPER tetramer (right panels), relative to background staining without tetramer (filled histograms). B cell populations in all tissues were gated as singlet, live, lineage^−^, CD19^+^B220^+^ cells, and subsets within B cell fractions were identified using published subfractionation schemes [44], [74]–[76]. Numbers indicate the percentage of tetramer-reactive B cells from a representative experiment (mean±s.d of five mice). B) Graphical representation of relative gp41 MPER reactivities in spleen (black) and peritoneal cavity (gray) B cell subsets, as calculated from several flow cytometry experiments (≥3 mice/experiment) as described in A). Numbers indicate the total mice analyzed.

To examine how gp41 MPER-reactive B cells were distributed among distinct B-cell compartments, we determined the frequencies of gp41 MPER-reactive B cells within splenic and peritoneal B cell subsets using two methods: by measuring the MFIs of distinct B cell subsets (Table S1) or by enumeration of B cells labeled by the gp41 MPER tetramer (Fig. 2 and Table S2). By both measurements, gp41 MPER reactivity was strongest in marginal zone (MZ) and peritoneal B1 B cell subsets of BALB/c mice (Fig. 2, Tables S1, S2), two B cell fractions expressing high levels of IgM (Fig. 2A and [44]) and restricted V_H_ family repertoires (45,46). In contrast, splenic mature follicular and peritoneal B2 B cell subsets, expressing lower levels of membrane IgM (Fig. 2A and [44]) and more diverse V_H_ repertoires [45], [46], exhibited lower levels of gp41 MPER binding. Examination of gp41 MPER reactivity in B-cell subsets of C57BL/6 mice did not reveal increased levels of MPER binding by MZ and B1a B cells (Table S2).

In addition to differing in overall frequencies and enrichment to particular subsets, gp41 MPER binding also differed qualitatively in BALB/c and C57BL/6 strains with respect to MFI binding patterns. In particular, the majority of MPER-reactive B cells within BALB/c subsets shifted as “low avidity” shoulders, with an additional, small fraction binding as a “higher-avidity” MFI tail (see Fig. 1A top right panel, 2A and MFI values in Table S1). In contrast, gp41 MPER-reactive C57BL/6 B cells scattered further out across the APC channel (Fig. 1A, top left panel, and Fig. 1C).

### Secreted IgM from in vitro activated BALB/c splenic B cells are enriched for gp41 MPER reactivity

**Figure 3:**
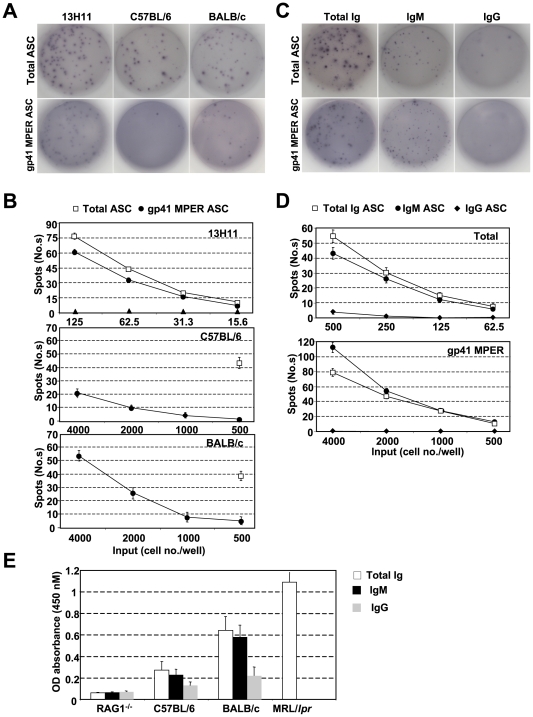
Analysis of gp41 MPER-specific ASCs from *in vitro*-activated splenocytes and gp41 MPER-specific serum Abs from naïve BALB/c and C57BL/6 mice. A) ASCs were measured by ELISPOT analysis of BALB/c or C57BL/6 splenocytes cultured for 72 h in LPS+BAFF, and the gp41 MPER-specific BALB/c hybridoma cell line, 13H11, for comparison. One of many replicate wells is shown for each panel. B) Graphical comparison of gp41 MPER-specific ASC frequencies in BALB/c and C57BL/6 cultured splenocytes. Shown are representative gp41 MPER-specific or dsDNA mimic-specific ASC values over a range of two-fold dilutions of 13H11 or input BALB/c or C57BL/6 splenocytes, all cultured in triplicate wells as described in A). The lowest cell dilution of total ASCs for each strain is also shown and was used to calculate the fraction of gp41 MPER-specific ASCs. C) Representative ELISPOTs of IgG or IgM-specificity within total and gp41 MPER-specific ASC from BALB/c splenocytes cultured as described in A). As a positive control for IgG-reactivity, also shown are IgG^+^ fractions within total and gp41 MPER-specific ASCs from13H11. D) Graphical representation of IgG, IgM, and IgM+IgG-specific fractions within total or gp41 MPER-specific ASCs, plotted as described in A). E) ELISA of C57BL/6 and BALB/c naïve serum reactivity to gp41 MPER. Naïve serum from five, 12 wk, female BALB/c and C57BL/6 mice were measured against plate-bound gp41 MPER, as described in Materials and Methods. Naïve serum from five, age and sex-matched RAG1^−/−^ and MRL/*lpr* animals (the latter exhibit significant gp41 MPER reactivity; Verkoczy and Haynes, unpublished results) were included as negative and positive controls, respectively.

To determine whether the high frequency, strain-dependent binding of gp41 MPER in IgM^hi^ B cell subsets was mediated by membrane IgM or some other strain-specific molecule, we activated splenocytes from naïve BALB/c and C57BL/6 mice *in vitro* with BAFF+LPS and measured the fraction of total (IgM+IgG) ELISpots that bound gp41-MPER peptide (Fig. 3). Consistent with the high frequencies of gp41 MPER^+^ BALB/c splenic B cells observed by flow cytometry (Fig. 1A,B), ∼17% of all BALB/c Ab-secreting cells (ASC) secreted IgM that bound gp41 MPER at a frequency 3 fold higher amount than C57BL/6 gp41 MPER-specific ASCs. Consistent with high frequencies of MPER-binding by IgM^hi^ B-cell subsets (Fig. 2 and Tables S1 and S2), ∼20% of IgM ELISpots were labeled by gp41 MPER peptide, whereas <5% of IgG ELISpots were gp41 MPER-specific (Fig. 3 C,D). Furthermore, at 500 input cells/well, ∼75% of total Ig, gp41 MPER-specific ASCs were IgM^+^.

Additional evidence for elevated, strain-dependent gp41 MPER reactivity came from spontaneous elevations in gp41 MPER-specific serum Ab from BALB/c mice, compared to C57BL/6 mice (Fig. 3E). This increase of gp41 MPER binding by BALB/c serum Ab was confirmed by Surface Plasmon Resonance (SPR) binding analysis (Fig. S2). Consistent with ELISpot data, serum Ab with the highest gp41 MPER reactivity were predominantly of the IgM isotype (Fig. 3E).

### HIV-1 gp41 MPER binding by naïve BALB/c B cells is epitope-specific

**Figure 4:**
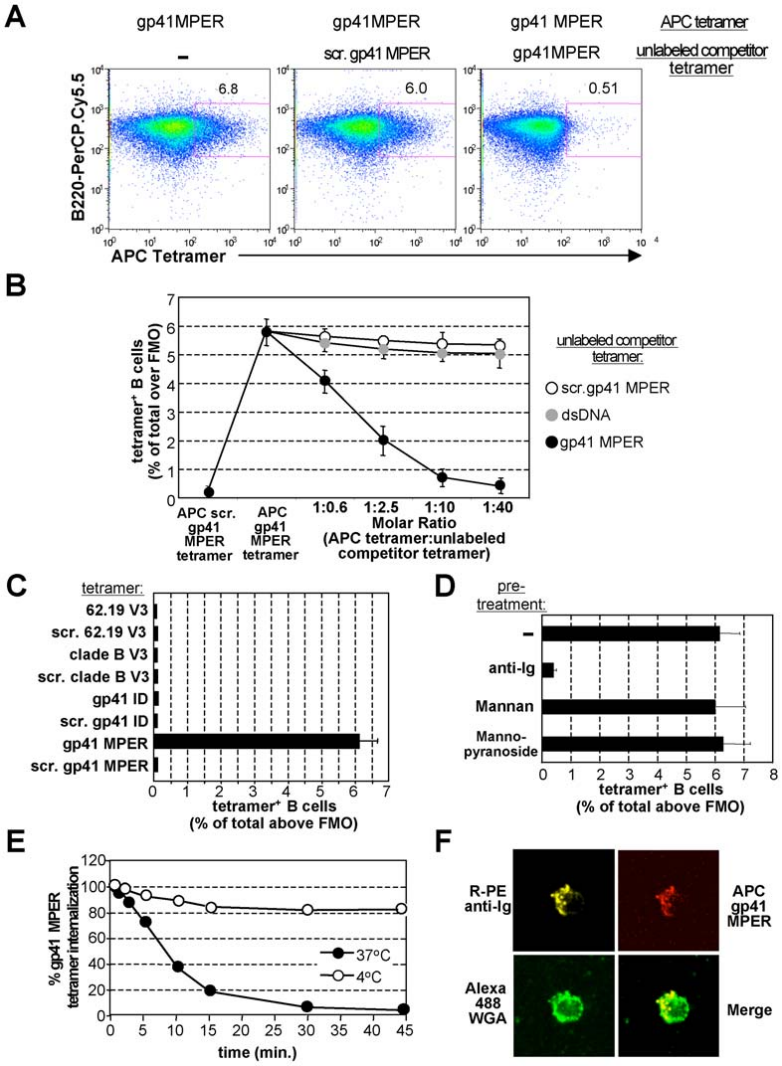
Specificity of gp41 MPER interactions with total B cells. A) Representative histograms showing cold inhibition of gp41 MPER binding to BALB/c B cells (100 ng/10^6^ splenocytes). Homologous competitor (unlabeled gp41 MPER tetramer) or heterologous competitor (scrambled gp41 MPER tetramer) were pre-incubated at 10-fold molar excess. Numbers represent the percentage of gp41 MPER-reactive B cells within singlet, live, CD19^+^ populations. B) Graphical representation of inhibition with increasing amounts of unlabeled gp41 MPER or heterologous (scrambled gp41 or dsDNA mimic) tetramer competitors. Data were calculated as in A) and represent the average of three experiments. C) Interactions of total BALB/c splenic B cells with various HIV-1 epitope-bearing B cell tetramers. Splenocytes were stained (100 ng tetramers/10^6^ cells) with gp120 V3, gp41 immunodominant, and gp41 MPER-specific B cell tetramers or their scrambled counterparts. Plotted are average percentages of tetramer-reactive B cells within singlet, live, CD19^+^ populations from three independent experiments. D) B cell tetramer reactivity in BALB/c splenocytes treated with BCR and MCLR ligands. 10^6^ splenocytes from naïve BALB/c mice were stained with 100 ng gp41 MPER tetramers, either alone, or pre-treated for 45 min at 37°C with anti-Ig, mannan, or α-methyl-mannopyranoside. Plotted are the percentages of tetramer-reactive B cells within singlet, live, CD19^+^ populations from an average of three experiments. E) Effect of BCR internalization on gp41 MPER surface staining of BALB/c splenic B cells. Anti-Ig stimulation of purified BALB/c splenic B cells for various times (in min.) and calculation of remaining surface-bound APC-labeled gp41 MPER was done as described in materials and methods; shown are results of a representative experiment. F) Cap formation of anti-Ig/gp41 MPER B cell tetramer complexes in BALB/c splenic B cells after BCR cross-linking, as described in materials and methods. An example of a capped B cell after 15 min of anti-Ig stimulation is shown, either as individual immunofluorescent stains of Alexa488 Wheat Germ Agglutinin, APC gp41 MPER tetramers, R-PE-anti IgM+IgG, or merged.

To exclude spurious, non-specific binding by gp41 MPER, we examined if binding was eliminated upon pre-incubating splenocytes with excess unlabeled gp41 MPER or scrambled gp41 MPER tetramers (homologous and heterologous competition, respectively) prior to labeling with APC-conjugated MPER tetramers (Fig. 4A). Pre-incubation with unlabeled gp41 MPER tetramer (but not scrambled tetramer) inhibited binding of the APC-labeled gp41 MPER tetramers >90%, confirming that gp41 MPER binding to BALB/c B cells was indeed epitope-specific.

We next pre-incubated BALB/c B splenocytes with increasing molar ratios of unlabeled gp41 MPER tetramers and two heterologous competitors: either i) unlabeled scrambled gp41 MPER or ii) a double-stranded DNA (dsDNA) mimic tetramer made from the R4A peptide DWEYSLWLSN [47], [48]. Dose-dependent inhibition of APC-labeled gp41 MPER peptide binding was observed with homologous competition, as measured by the percentage of gp41 MPER^+^ B cells (Fig. 4B) or by MFI (data not shown). Importantly, incubation of dsDNA tetramers showed minimal competition with gp41 MPER binding, indicating that any potential dsDNA-specific BALB/c B cells were unrelated to those specific for gp41 MPER.

We then asked if this unusual, high-frequency (≥5%) binding to BALB/c B cells was unique to gp41 MPER. To do this, we made tetramers from peptides spanning conserved epitopes of two other well-characterized HIV-1 Env regions, the V3 and immunodominant (ID) loops of gp120 and gp41, respectively, and tested the ability of these reagents to stain naïve BALB/c splenocytes. The specificity/quality controls of the gp120 V3 and gp41 ID-specific tetramer reagents are shown in Fig. S1. We chose these two HIV-1 Env regions to compare with gp41 MPER because they are conserved, conformationally accessible, and known to be immunogenic i.e. capable of eliciting robust epitope-specific Ab responses [49], [50]. We reasoned that if gp41 MPER tetramer binding to B cells represents an inherently unique feature of the gp41 MPER epitope (rather than specificity and/or avidity issues exhibited by all HIV-1 epitope-specific tetramer reagents), then gp41 ID and V3-specific tetramers should bind B cells at frequencies seen by conventional antigens. In response to immunization, such clonal, antigen-specific binding occurs at relatively low-frequencies i.e. 0.01–1% (47), and in unimmunized mice, likely occurs at frequencies several orders of magnitude lower yet. Indeed, when we incubated naïve BALB/c splenocytes with either of the two V3 tetramers, (62.19 V3 and clade B V3), or with the gp41 ID-specific reagent, no tetramer reagent other than gp41 MPER labeled B cells at frequencies significantly higher than their respective scrambled tetramer controls (all scrambled controls bound with background levels of ∼0.1–2% of total B cells; Fig. 4C). We thus conclude that the high-frequency binding of BALB/c B cells to gp41 MPER is specific for, and unique to this epitope.

### The gp41 MPER epitope interacts with large fractions of BALB/c B cells by specifically binding to B cell receptors (BCRs)

The frequencies of BALB/c B cells and secreted, IgM ELISpots that bound gp41 MPER peptide was much higher than expected for any antigen-specific population. Although the ELISpot data indicated that MPER peptide binding was mediated by surface IgM, we examined whether gp41 MPER could interact with BALB/c B cells via other surface receptors such as Mannose C type Lectin Receptors (MCLRs; through which gp120 has been reported to interact with [38]). We first examined relative effects of BCR and MCLR ligands to block and/or internalize gp41 MPER (Fig. 4D). APC gp41 MPER signal was significantly abrogated following incubation with an anti-Ig, but not with MCLR ligands mannose and alpha-methyl-mannopyranoside, suggesting specificity of gp41 MPER for BCR.

We next determined if anti-Ig mediated downmodulation of gp41 MPER binding was due to internalization of gp41 MPER/Ig complexes by pre-incubating with anti-Ig Abs (Fig. 4E). MPER peptide downmodulation with anti-Ig at 37°C was rapid (>80% by 15 min), and was not observed at 4°C, suggesting that BCR internalization accounted for diminished gp41 MPER binding. To further determine whether bound gp41 MPER associated with membrane Ig, we performed co-capping experiments (Fig. 4F). Indeed, gp41 MPER tetramer co-capped with surface Ig in BALB/c splenic B cells stimulated with both reagents. Collectively, our results demonstrate that gp41 MPER interacts with large fractions of naïve BALB/c B cells by binding to membrane-associated Ig.

### High frequency gp41 MPER binding to naïve B cells maps to the Igh^a^ locus

Our results demonstrating strain-specific interactions of gp41 MPER with large fractions of naïve B cells through membrane IgM raised the possibility that gp41 MPER does not bind to V_H_ regions in conventional antigen-specific fashion, but instead, binds allotypic residues in conserved V_H_ framework and/or C_H_ domains.

**Figure 5:**
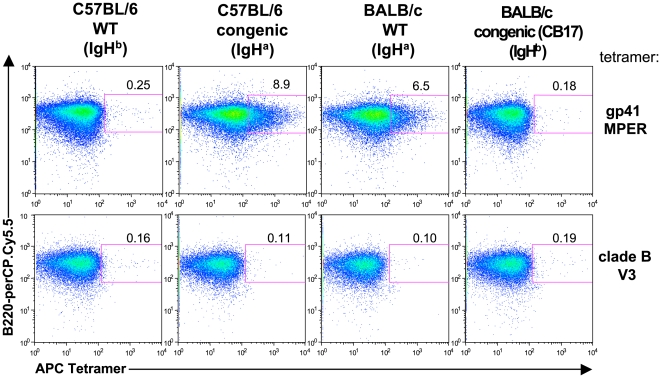
Analysis of IgH allotype involved in gp41 MPER epitope interactions with the BCR. Representative histograms showing staining of 10^6^ splenocytes from unimmunized C57BL/6 and BALB/c inbred strains (congenic for IgH^a^ and IgH^b^ allotypic determinants) with 100 ng of gp41 MPER or clade B V3-specific APC-labeled tetramers. Numbers indicate the percentage of tetramer-reactive cells within singlet, live, CD19^+^-gates. Results are representative of several experiments; similar results were seen in B cells from BM, LN, and PBL (not shown).

To determine whether the frequent gp41 MPER binding by BALB/c B cells is controlled by the Igh^a^ locus, we determined the frequencies of B cells binding gp41 MPER tetramer in congenic mouse strains differing solely at their Igh loci (Fig. 5). Control tetramers carried the epitopes of clade B V3. Strikingly, we found that high frequency gp41 MPER binding segregated with the Igh^a^ allele, as C57BL/6 mice homozygous for the BALB/c Igh^a^ locus exhibited gp41 MPER binding identical to BALB/c animals (Fig. 5). Conversely, congenic BALB/c mice homozygous for the C57BL/6 Igh^b^ locus lost gp41 MPER binding (Fig. 5).

### High frequency, Igh^a^-restricted gp41 MPER binding maps to non-paratopic regions of IgM

Preferential interaction of gp41 MPER with IgM-expressing B-cell subsets (Fig. 2, Tables S1, S2) and secreted IgM (Fig. 3B), along with genetic demonstration of Igh^a^-restricted binding (Fig. 5) strongly imply that gp41 MPER binds to naïve B cells via IgM^a^-restricted determinants. Since interactions can occur between BCRs and B-cell superantigens (sAg) through V_H_ framework (FRW) or Fc (reviewed in [37]), we examined where gp41 MPER peptide bound to IgM^a^ molecules.

**Figure 6:**
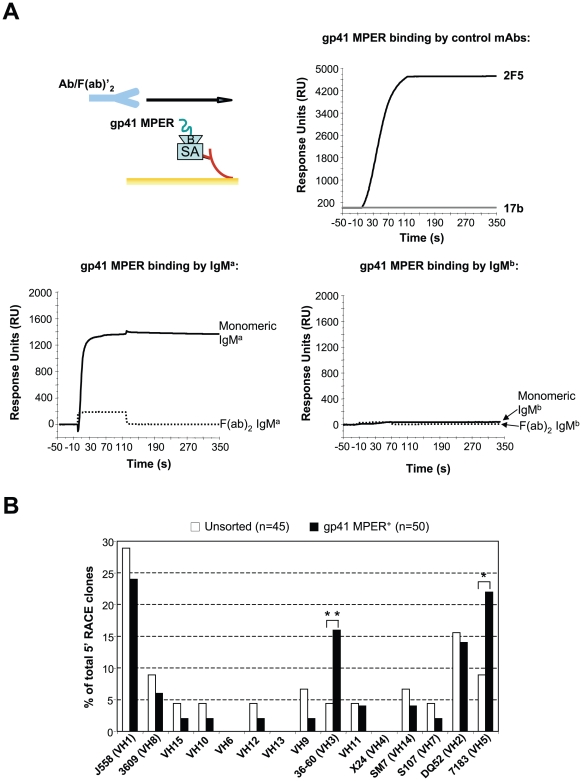
Mapping of IgM regions required for allotype-restricted binding to gp41 MPER. A) Surface plasmon resonance analysis of IgM^a^ and IgM^b^ binding to gp41 MPER. Monomeric or F(ab)_2_ fragments derived from IgM^a^ and IgM^b^ mAbs were injected over biotinylated (B) gp41 MPER nominal epitope peptide, anchored to a SA-coated sensor chip, as shown in the top left-hand schema, and described in materials and methods. mAbs 17b and 2F5 were run as negative and positive controls for gp41 MPER binding, respectively. Results are representative of two independent experiments. B) V_H_ family usage in gp41 MPER-sorted IgM^+^ B cells from naïve BALB/c mice. Independent 5′ RACE clones (n) were derived from purified BALB/c splenic B cell populations, either unsorted or sorted into gp41 MPER^+^ fractions and subjected to IgM-specific 5′ RACE analysis, as described in Materials and Methods. V_H_ family frequencies within expressed V_H_ repertoires was determined by Ig Blast analysis of sequences. Statistical comparisons between unsorted and gp41 MPER+ fractions were performed using the Fisher Exact test (*, p<0.05; **, p<0.01). Results are pooled from multiple PCR amplifications of two sorts. V_H_ families are represented in the 5′ to 3′ order by which they map to the V_H_ locus.

First, we performed surface plasmon resonance analyses of gp41 MPER binding to whole, monomeric or F(ab)_2_ IgM fragments of two purified IgM mAbs, differing in allotype (IgM^a^ and IgM^b^), but with the same antigenic specificity to TNP-KLH. We first examined binding of monomeric IgM^a^ and IgM^b^ to gp41 MPER (Fig. S3). As expected, preferential binding with IgM^a^ was observed: this binding was specific for the gp41 MPER 2F5 epitope, as the scrambled gp41 MPER peptide or a peptide corresponding to a region within gp41 MPER, but outside the 2F5 epitope (gp41 MPER HR1) exhibited little or no binding. This binding reflected low avidity interaction(s), ∼1 log lower avidity than did 2F5-gp41 MPER interactions (Kd values were 275.5 and 10 nM, respectively), as calculated by surface plasmon resonance analysis (Table S3). Importantly, comparison of gp41 MPER binding by F(ab)_2_ IgM^a^ fragments or by whole, monomeric IgM^a^ revealed that F(ab)_2_ IgM^a^ did not bind the gp41 MPER epitope (Fig. 6A). This loss of binding to the gp41 MPER by F(ab)_2_ IgM^a^ fragments was unlikely due to loss of functionality/conformational integrity since their TNP specificity was retained (Fig. S4). We conclude that allotypic determinants in the IgM^a^ constant region are critical for the binding of gp41 MPER to BALB/c B cells.

These studies, as well as the fact that MZ and B1 cells, in addition to expressing higher IgM levels, also contain more restricted V_H_ repertoires [45], [46], [51], cannot exclude the possibility of Igh^a^-linked variable region determinants, for example within the FRW of selected V_H_ families that may also contribute to gp41 MPER binding. To test this possibility, we determined the pattern of V_H_ family usage in the expressed IgM^+^ V_H_ repertoire of gp41 MPER tetramer-sorted splenic BALB/c B cells (Fig. 6B). Compared to total IgM^+^ BALB/c B cell populations, we found that most V_H_ families were utilized at statistically comparable levels in gp41 MPER-reactive B cells, with the exception of two overutilized V_H_ families: 36–60 and 7183. From this analysis, we conclude that allotype-restricted gp41 MPER interactions with IgM^a^ require Fc C_H_ domains, but that determinants of the V_H_36-60 and −7183 families may also play a role.

### Igh^a^ restricted binding of gp41 MPER at residues distinct from those required for bnAb 2F5 binding/neutralization

**Figure 7:**
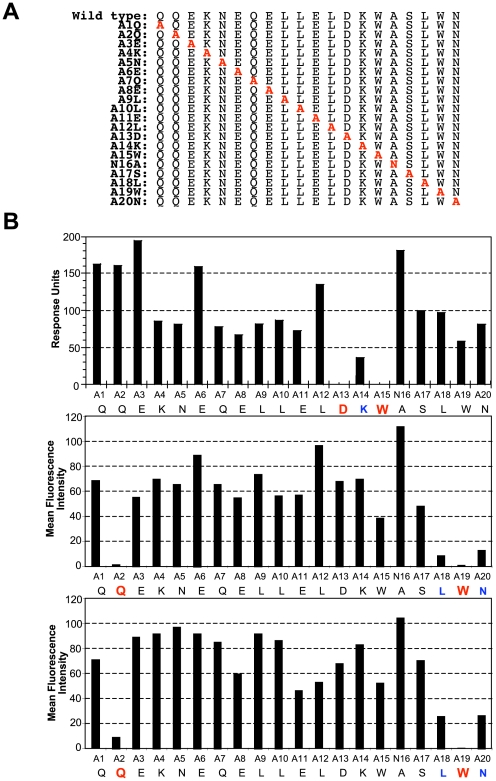
Mapping of gp41 MPER residues required for high frequency interactions with IgM^a^. A) List of peptides (annotated numerically according to position of substitution) used to make mutant tetramers with sequential alanine substitutions (in red) along the gp41 MPER region containing the nominal 2F5 epitope. B) Comparison of gp41 MPER residues required for binding bnAb 2F5 with those required for allotypic interactions with IgM. Shown is relative binding of mutant tetramers with either 2F5 (upper panel), naïve BALB/c total splenic B cells (middle panel), and 9G8 (lower panel). Tetramers with corresponding substituted positions in the 2F5 epitope are indicated on the x-axis, with those in blue and red representing mutated residues with >3 and >10 fold binding reductions, respectively. For measuring mutant tetramer binding to 2F5, unlabeled versions of tetramers were used in SPR analysis, as described in Materials and Methods. For measuring binding of mutant tetramers to 9G8 and to naïve BALB/c B cells, APC-labeled tetramers were used for staining splenocytes (100 ng/10^6^ cells), followed by calculating APC MFIs of singlet, live, total B cells after subtracting background MFIs in unstained cells. Data shown are an average of two experiments.

To determine which aa residues in the gp41 MPER epitope mediate high frequency, low affinity binding to IgM^a^ B cells, we created a series of MPER peptide variants by alanine substitution (Fig. 7A) and compared their binding of gp41 MPER with 2F5 (Fig. 7B, top panel) to binding with BALB/c splenic B cells (Fig. 7B, middle panel). Consistent with previous studies [18], [20], we found that binding of 2F5, required residues 671D and 673 W within the core 2F5 neutralization epitope ELDKWA (Fig. 7B, top panel). Interestingly, gp41 MPER binding to BALB/c B cells mapped to 660Q and a 677 W, both located outside the core 2F5 epitope (Fig. 7B, middle panel).

To extend this result, we generated splenic fusions from naïve IgH^a^ and IgH^b^ congenic BALB/c mice and screened them for gp41 MPER reactivity by flow cytometry using gp41 MPER tetramers. Consistent with the IgM^a^ requirement for gp41 MPER binding, we did not identify gp41 MPER-reactive IgM^b^, or IgG^a^ or IgG^b^ clones; we did, however, identify four gp41 MPER-reactive IgM^a^ hybridoma lines (data not shown). Of these, we used the cloned hybridoma, 9G8, with the highest gp41 MPER reactivity (as determined by MFI), to map gp41 MPER-IgM^a^ interactions (Fig. 7B, lower panel). Strikingly, we found that the 660Q and 677W residues required for binding BALB/c splenic B cells were also critical for binding to 9G8 cells. Since the 671D and 673W residues of gp41 MPER interact with HC CDR regions from the 2F5 antigen-binding pocket, our finding that different gp41 MPER residues are required for IgM^a^-specific interactions is consistent with such interactions occurring in IgM F_c_ or possibly, V_H_ FRWs.

### gp41 MPER tetramerized peptides can induce partial B cell activation in vitro

**Figure 8:**
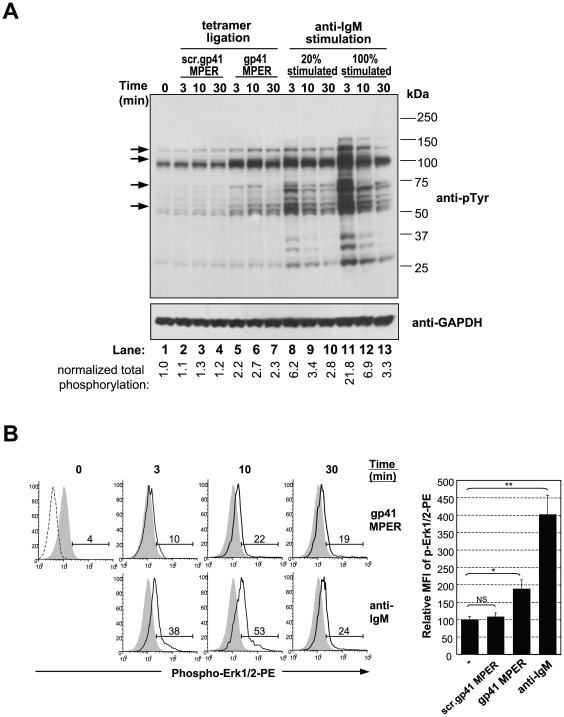
Analysis of proximal B cell signaling/activation events in response to ligation with gp41 MPER. A) Purified splenic B cells from naïve BALB/c mice, either incubated with medium alone (time = 0) or stimulated with 20 µg/ml scrambled gp41 MPER tetramer, gp41 MPER tetramer, or F(ab)'_2_ anti-IgM for indicated times were assessed for total phosphotyrosine proteins by immunoblotting (top panel). Lower panel shows the same blot, re-probed with a GAPDH-specific Ab. Selectively activated proteins in response to gp41 MPER ligation are indicated by arrows and values below lane numbers indicate relative phosphorylation levels normalized to anti-phosphotyrosine/GAPDH signal ratios in resting (time = 0) cells. B) Left: Intracellular FACS staining analysis of phosphorylated Erk levels in naïve BALB/c splenocytes, either unstimulated (filled histograms) or stimulated for 3–30 min (open histograms) with 20 µg/ml APC-labeled gp41 MPER tetramer (top panel) or 10 µg/ml APC-labeled anti-IgM or (lower panel). Data are gated on B220+IgM (for anti-IgM stimulated samples) or on B220+tetramer (for gp41 MPER-stimulated samples). The upper left hand panel shows Erk signal levels in resting cells relative to staining with an isotype control (dotted line). Numbers indicate the percentage of cells with phosphorylated Erk. Right: Relative Mean Fluorescence Intensity (MFI) of phospho-Erk1/2-PE signal +/− SD from three experiments in which naïve BALB/c splenocytes were either left unstimulated (−) or stimulated for 10 min. with 10 µg/ml anti-IgM or scrambled gp41 MPER/gp41 MPER tetramers. Mean levels of phosph-Erk1/2-PE in unstimulated splenocytes were arbitrarily set at 100. Significance values were determined by a two-tailed Student's test: *, p≤0.05; **, p≤0.01; NS, not significant.

To determine if gp41 MPER interactions with IgM^a^ could elicit the activating signals required for biological responses, we assessed BCR-proximal signaling events in BALB/c splenic B cells ligated with gp41 MPER tetramers. First, we determined the induction of protein tyrosine phosphorylation in cells incubated with saturating concentrations (20 µg/10^6^ cells; see Fig. 1b) of gp41 MPER tetramer (Fig. 8A). Under the assumption that ∼20–25% of the total B cells bind saturating amounts of gp41 MPER tetramers, tyrosine phosphorylation was assessed in lysates containing a 4∶1 mixture of resting:anti-IgM stimulated BALB/c splenic B cells. gp41 MPER tetramers induced rapid and reproducible patterns of protein phosphorylation (Fig. 8A, lanes 5–7), albeit in quantities 2- to 3-fold lower than that generated from anti-IgM ligation of equal numbers of B cells (Fig. 8A, lanes 8–10). In addition, only selected proteins were phosphorylated, including Syk, PI3K p110δ, PLC-γ2, and one or more of the Src kinase family members Lyn, Fyn, or Blk, (putatively represented by 72, 110, 130, and 56–60 kDa bands, respectively). Importantly, incubation of B cells with scrambled gp41 MPER tetramer did not induce protein phosphorylation above background levels (Fig. 8A, lanes 2–4).

To distinguish whether all B cells that bound gp41 MPER tetramers signaled weakly, or alternatively, if a small B-cell subset was preferentially activated, we measured Erk protein phosphorylation in BALB/c splenic B cells exposed to gp41 MPER or anti-IgM by intracellular FACS analysis (Fig. 8B). Because this approach allowed us to gate anti-Ig or gp41 MPER peptide-ligated B cells, the amount of anti-Ig and gp41 MPER-induced Erk phosphorylation could be compared on a cell-per-cell basis. Consistent with phosphoblotting analyses, detectable, but dampened induction of phosphorylated Erk was seen with gp41 MPER stimulation (Fig. 8B, top panels) relative to anti-Ig ligation (Fig. 8B, lower panels). Howver, Erk phosphorylation kinetics differed between gp41 MPER and anti-Ig stimulation. Specifically, peak gp41 MPER-induced Erk phosphorylation persisted at 30 min, whereas anti-Ig induced Erk phosphorylation peaked by 10 min. Importantly, the entire population of gp41 MPER-gated B cells showed modest Erk activation, suggesting that all gp41 MPER-ligated cells were equivalently activated.

Overall, these results demonstrate that gp41 MPER can generate BCR, but relative to anti-Ig stimulation, these elicit weak and/or partial BCR-proximal signaling patterns. We conclude that gp41 MPER interactions with IgM^a^ may produce signals distinct from those elicited by higher-affinity, paratopic BCR cross-linking.

## Discussion

In this study, we show that the 2F5 epitope of the HIV-1 gp41 MPER interacts with BALB/c MZ and B1 IgM^a^ B cells in a manner that is distinct from traditional, clonal (paratopic) binding by IgM^b^ B cells. This unusual binding is also distinct from the interaction of other HIV-1 Env epitopes with IgM^a^ or IgM^b^ B cells. These non-paratopic interactions are frequent (Fig. 1B) and exhibit remarkable specificity, as demonstrated by ELISpot analysis (Fig. 3), cross-epitope competition assays (Fig. 4A,B) and biochemical/immunohistochemical evidence of specificity for sIg (Fig. 4D–F). Secondly, our data demonstrate that these interactions occur between IgM^a^ allotypic determinants not present on F(ab)_2_ fragments (Figs. 5,6) and involve gp41 MPER residues 660Q and 677W (Fig. 7), distinct from those known to be critical for bnAb 2F5 neutralization. Thirdly, gp41 MPER-IgM^a^ interactions are low avidity, as determined by: i) i) SPR binding measurements of gp41 MPER with IgM^a^ (Table S3) and ii) elicitation of weak/incomplete proximal B cell activation events by gp41 MPER (Fig. 8).

One obvious question arising from this study is whether gp41 MPER exhibits B cell sAg activity. Classical B-cell sAgs, like the S. aureus protein A subunit (SpA), can bind to and activate large populations of B cells, may cause selective deletion of MZ and B1 B cell subsets, and bind to B cells based on V_H_ family usage [37], [52]. Similarly, sAg interactions of HIV-1 gp120 with human B cells have been described (reviewed in [37] and [53]–[56]), resulting in a V_H_3-skewed repertoire in HIV-1 infected individuals (reviewed in [57] and [58]–[60]). Characteristics of gp41 MPER binding to IgM^a^ described in this study that are comparable to SpA-BCR or HIV gp120 sAg include: 1) the large fraction of total B cell populations bound by gp41 MPER via BCR HCs, 2) preferential binding to MZ and B1 B cell populations 3) non-paratopic binding of gp41 MPER 4) partial deletion of MZ and B1 B cells in gp41 MPER-immunized BALB/c mice (L. Verkoczy and B. Haynes, unpublished results).

Our data suggest that non-clonal binding of gp41 MPER to B cells requires IgM C_H_ (Fig. 6A), providing a straightforward explanation for why a large portion of MZ B cells have uniformly higher gp41 MPER reactivity (Fig. 2A and Table S1) than MF B cells (which have comparable sIg densities, but lower IgM expression). While the specificity of gp41 MPER for C_H_ is not shared by HIV gp120 sAg binding, it is compatible with SpA, which was first characterized in the context of its interactions with Fc regions [61], [62]; subsequently SpA was shown to have V_H_ FRW-binding properties [62]. Another intriguing feature of gp41 MPER interactions with B cells is the segregation of binding with IgM^a^ determinants (Figs. 5, 6). Interestingly, this characteristic is shared with T15, a dominant, SpA-reactive, anti-phosphorylcholine (PC) idiotope on B1 B cells, whose allotypic determinants are key in shaping the anti-PC B cell repertoire and humoral response [63], [64]. Immunization/neonatal challenge studies with gp41 MPER in BALB/c and C57BL/6 IgH congenic strains are currently underway to determine if/how gp41 MPER might exhibit sAg activity on B cell differentiation and development.

It is also interesting that gp41 MPER reactive B cells preferentially use the proximal V_H_ families 36–60 and 7183. While this could simply be correlational i.e. due to innate IgM^hi^ B cell subsets also overutilizing proximal V_H_ families (for example, skewing of V_H_7183 in B1a cells; [65]), an intriguing alternative is that IgM^a^ allotypic determinants in 7183 and 36–60 V_H_ FRW regions (in combination with C_H_ determinants), may form a gp41 MPER-specific idiotope, a situation analogous to the T15 sAg-reactive idiotope [63]. Future genetic experiments using BAB/14 congenic mice [66], with a recombination between V_H_ and C_H_ allotypes (i.e. having an Igh^a^ (BALB/c) V_H_ region and an Igh^b^ (B6) C_H_ region) should definitively address any potential, additional contribution of the V_H_ region in the non-clonal binding of Igh^a^ determinants to the gp41 MPER.

An alternative possibility for preferential gp41 MPER interactions with IgM^a^ allotypic determinants is that determinants within (or proximal to) the Igh^b^ locus may encode a self-antigen mimicked by gp41 MPER. This situation would be analogous to MMTV infection-susceptible congenic mouse strains, which retain endogenous MMTV-like sAg genes that delete sAg-binding T cells with the same Vβ specificities as those encoded by the infectious virus during the shaping of the TCR repertoire (reviewed in [67]). Assuming gp41 MPER sAg IgM^a^-specific binding were due to the lack of IgM^b^ endogenous allotype-encoded determinants, the differential binding patterns to gp41 MPER in BALB/c and C57BL/6 B cell subsets may therefore reveal an inherent susceptibility of the endogenous gp41 MPER-specific B cell repertoire to: i) an IgM^b^ allotype-specific endogenous sAg (that cross-reacts with gp41 MPER), reflected by the uniformly higher sAg-mediated binding across all IgM^a^ subsets, and ii) the same or a different IgM^b^-specific endogenous sAg selectively targeting innate subsets, reflected by further preferential elevations in IgM^a^ MZ and B1 subsets. We are currently generating transgenic Igh^a^ and Igh^b^ congenic mice overexpressing gp41 MPER to address these possibilities.

The demonstration that gp41 MPER interactions with IgM^a^ allotypic determinants outside antigen binding pockets are capable of eliciting BCR-proximal activation signals makes the important link between this unusual binding and its potential impact on gp41 MPER-specific humoral responses. In this context, one obvious question arising from our studies is what are the physiological consequences of weak and/or incomplete signaling by gp41 MPER (Fig. 8). One possibility is that incomplete activation of B cells by low affinity MPER binding could suppress the T-independent component of MPER Ab responses, as has been reported for low-affinity, weak/partial agonists [68]. This potential lack of IgH^a^ T-independent anti-gp41 MPER responsiveness could be enhanced by efficient T-B collaborations *in vivo* due to preferential recognition of certain T_H_ cells for IgH^a^, since there is precedent for T cell-specific recognition of IgH^a^ C regions [69], [70]. Two additional possibilities related to avidity and frequency of MPER-IgM^a^ interactions, (but functionally independent of the interacting allotypic determinants), could be invoked. First, incomplete weak or incomplete BCR signaling by low affinity MPER binding could deliver anergic signals in gp41 MPER-interacting peripheral B cells, thus blocking an antigen-specific response through BCR, analogous to antagonistic signals delivered by low affinity BCR and TCR ligands. Secondly, B cell subsets with different signaling thresholds could be differentially modulated by MPER. In particular, because MZ B cells (relative to MF B cells) require weaker BCR signals [71]–[73], and are more sensitive to anti-IgM mediated apoptosis [51], the large fraction of MPER-specific MZ B cells in BALB/c mice may be preferentially susceptible to deletion via MPER ligation through BCR, a possibility that would be consistent with reduced MZ B cell populations in gp41 MPER-immunized BALB/c mice (Verkoczy and Haynes, unpublished results).

Regardless of the signals that interactions between gp41 MPER and IgM^a^ generate, we propose that distinct BCR interactions with gp41 MPER exist in BALB/c and C57BL/6 mice that may reflect two distinct immunoregulatory mechanisms controlling MPER-specific bnAb responses: 1) high-affinity, low-frequency, developmentally-regulated patterns of “antigen-specific” gp41 MPER binding in C57BL/6 mice (M.Holl, L.Verkoczy, B.Haynes, and G.Kelsoe, unpublished data) presumably involving paratopic interactions with long, hydrophobic HC CDR3 regions of 2F5 or “2F5-like”-expressing B cells, and 2) “sAg-like” binding in BALB/c mice in this study, representing high frequency, low-affinity non-clonal interactions of IgM^hi^ B cell subsets with gp41 MPER, capable of eliciting sub-optimal and/or altered B cell signaling. However altered or dampened signals generated by the gp41 MPER may interfere with an effective humoral response to this region (i.e. either triggering a robust non-neutralizing MPER Ab responses and/or eliciting poor bnAb anti-MPER responses), the finding that unique gp41 MPER residues are involved in such interactions may provide clues for immunogen design. Specifically, designing gp41 MPER immunogens that abrogate allotype-regulated MPER binding may yield immunogens with only antigen-specific B cell activation capabilities.

## Materials and Methods

### B cell tetramer synthesis and validation

N-biotinylated, linker/spacer-containing peptides used to make tetramers are detailed in Table S4 and Fig. 7A. Peptides were synthesized and purified using reverse-phase HPLC (Primm Biotechnology). To produce tetramerized forms of each peptides, 200 µM peptide and 6 µM APC-labeled streptavadin (SA) were combined at equal volumes, and mixtures were incubated at 4°C for 4 h. Unbound peptide was removed from peptide-APC complexes by centrifugal filtration using an Amicon Centriprep YM30 column (Millipore Corporation). Purified tetramer preparations were determined using the Micro BSA protein assay kit (Pierce Biotechnology).

### Mice and gp41 MPER-specific hybridomas

Female C57BL/6, BALB/c, B6Igh^a^, and CB17 (BALBc Igh^b^) inbred mouse strains (8–12 wks of age) were purchased from Charles River Laboratories. All mice were housed in the Duke University Animal Facility in a pathogen-free environment with 12 h light/dark cycles at 20–25°C under AALAC guidelines and in accordance with all Institutional Animal Care and Use Committee and Duke University Institutional Biosafety Committee-approved animal protocols. The IgG1 anti-gp41 MPER cell line 13H11 was grown and maintained in DMEM media (Gibco) containing 10% FCS, 10^−4^ M 2-ME and penicillin/streptomycin (P/S) antibiotics as previously described [39]. The 9G8 IgM^a^ anti-gp41 MPER hybridoma cell line was generated from a hybridoma fusion performed using P3X63/Ag8 murine myeloma cells and spleen cells taken from an unimmunized BALB/c mouse.

### In vitro cultures and ELISpot

ELISpot plates (Millipore) were coated with 2 µg/ml (50 µl/well) of goat anti-mouse Ig(H+L), goat anti-mouse IgM or goat anti-mouse IgG in 0.1 M Carbonate Buffer (pH 9.5) overnight at 4°C. Washing/Blocking buffer contained 1x PBS (pH 7.4), 0.1% Tween-20 and 0.5% BSA (USB). For detection of antigen-specific ASC, LPS+BAFF-activated B cells were washed and plated at 0.5−4×10^3^ cells/well in triplicate. Cells were incubated at 37°C in a humidified CO_2_ incubator for 4 h with IMDM media containing 10% FCS, 10^−4^ M 2-ME and penicillin/streptomycin. Plates were washed and re-blocked for 1-2 d using blocking buffer, and membranes were probed with 20 µM biotin-DP178Q16L peptide (YTSLIHSLIEESQNQQEKNEQELLELDKWASLWNWF; containing the gp41 MPER 2F5 epitope), for 2 h at room temperature. SA-AP (Southern Biotech) and SIGMA FAST BCIP/NBT (Sigma) were used to develop antigen-specific spots. For detection of total ASC, LPS+BAFF-activated B cells were plated, washed and re-blocked as described above, and membranes were detected with goat-anti-mouse IgM-AP and IgG-AP.

### ELISA

For determination of gp41 MPER-specific serum Ab titers, ELISA was performed by coating high-binding microtiter plates with the peptide QQEKNEQELLELDKWASLWN; (corresponding to residues 659–678 of the HIV-1 envelope and containing the optimal, nominal gp41 MPER 2F5 epitope; 0.2 µg/well) as previously described [40].

### Flow cytometry

FACS staining of single cell suspensions (≥2×10^6^) was conducted using pre-mixed combinations of fluorochrome-labeled Abs and APC-conjugated B cell tetramers at empirically-determined optimal concentrations. All Abs were from BD unless otherwise stated. Primary antibodies used were: PerCP anti-B220, FITC anti-IgD, FITC, PE or PE-Cy7 anti-IgM, PE anti-CD5, FITC anti-CD11b, PE-Cy7 anti-CD19, FITC anti-CD21, PE-Cy7 anti-CD23 (eBiosciences), and PE-Cy7 anti-CD93 (eBiosciences). Other reagents included biotinylated Abs against dump channel markers: Thy1 (Abcam), F4/80 (Abcam), CD11c, Gr-1, TER-119, and NK-1.1, secondary staining SA-Texas Red conjugate; v-amine live/dead violet dye (Molecular Probes), and Fc block (anti-CD16/32).

Staining was conducted at 4°C in several sequential steps in FACS staining buffer (1X PBS with 2% FCS, 0.1% NaN_3_), washing twice between steps. Cells were first incubated with v-amine live/dead dye for 15 min, then stained with APC-labeled tetramers for 30 min (and in some experiments, cold tetramer was incubated at 10 fold molar excess for 1 h, prior to staining with APC-labeled tetramers). Cells were then stained with Fc block, followed by incubation with combinations of primary Abs, and secondary staining with SA-Texas Red, each for 30 min. Data (≥2.5×10^5^ events/sample) were acquired using an LSRII flow cytometer and Cell Quest software (BD Immunocytometry systems). FACS Analysis was performed using FlowJo software (Tree Star). For analysis, cells were gated in the following order: single cells were gated based on fsc height vs. fsc area+ssc height vs. ssc profiles, lymphocytes were gated on fsc and ssc profile, residual dead cells were excluded by negative gating with v-amine live/dead dye, and irrelevant non-B cells were excluded by negative gating with dump markers.

### Cell sorting and 5′ Rapid Amplification of cDNA Ends (5′ RACE)

For sorting of gp41 MPER tetramer-binding B cells, single cell splenic suspensions were obtained from 5–10 naïve BALB/c 8 wk females, pooled, and purified by negative depletion with CD43 MACS beads (Miltenyi Biotechnology). The resulting B cell enriched fraction was stained as described in the flow cytometry methods section and sorted into B220^+^ CD19^+^ gp41 MPER^+^ fractions using a BDFACS Aria. Sorting gates for the gp41 MPER^+^ fractions were set to include all events greater than baseline staining without tetramer. Purity of sorted populations was verified by a 1000-event post-sort step.

For 5′ RACE analysis, gp41 MPER^+^ fractions (or purified, unsorted splenic B cells) were spun down and washed, immediately after sorting. DNAse-free RNA was extracted and concentrated using RNEasy Mini and Micro kits, respectively (Qiagen) and quantitated using a NanoDrop ND-1000 spectrophotometer (Fisher). cDNA was synthesized by oligo-dT priming using SuperScript III (Invitrogen) and IgM-specific 5′ RACE products were amplified with the First Choice RLM-RACE PCR kit (Ambion), using mouse HC̃-specific primers in combination with 5′ RACE primers, according to the manufacturer's instructions. PCR products were cloned into pGEM-T Easy vectors (Promega), transformed in GC5 competent cells (CLP), and transformant DNA was isolated, sequenced and analyzed in Ig Blast.

### BCR internalization assays

Splenic B cells, purified by negative depletion with CD43 MACS beads, were pre-incubated on ice for 30 min with a 95∶5 unlabeled/R-PE-labeled mixture of 10 µg/ml goat anti-mouse-IgM+IgG (H+L) F(ab)_2_ fragments (Jackson ImmunoResearch Laboratories). After washing, cells were resuspended in DMEM 10% FCS, and stimulated by warming to 37°C (or at 4°C as a control) for the indicated time points. Cells were fixed immediately (for t = 0) or at indicated time points with 1% methanol-free formaldehyde PBS solution, stained with PerCP-B220 and either APC-labeled gp41 MPER or scrambled gp41 MPER tetramers, and detected by flow cytometry. Percent tetramer internalization was calculated in gp41 MPER+B220+ subsets by the formula [% gp41 MPER (T_o_) -% gp41 MPER (T_n_)]/%Sp62 (T_o_) X 100. In other experiments, cells were pre-incubated with 5 mg/ml mannan, 125 mM α-methyl-mannopyranoside (Sigma-Aldrich), or 10 µg/ml unlabeled goat anti-mouse-IgM+IgG (H+L) F(ab)_2_ fragments prior to staining with APC-labeled tetramers.

### Microscopy of BCR cap formation

Purified naïve splenic B cells were incubated on ice for 30 min with Alexa 488-labeled wheat germ agglutinin (Invitrogen) and 10 µg/ml R-PE-labeled goat anti-mouse IgM+IgG (H+L) F(ab)_2_ and APC-labeled gp41 MPER. After extensive washing, cells were stimulated for indicated times. Images were acquired with a Nikon TE2000-E2 inverted microscope employing a 40X magnification lens from a CoolSNAPPHQ2 monochrome camera.

### Intracellular staining

Splenocyte suspensions were incubated at 37°C for various times with either APC-labeled F(ab)'_2_ anti-IgM (Jackson ImmunoResearch) or APC-labeled gp41 MPER tetramers. Stimulations were stopped by the addition of PhosFlow Lyse/Fix buffer (BD) and incubation for 10 min at 37°C. Cells were stained with B220-FITC prior to permeabilization with PhosFlow Perm Buffer III (BD). Permeabilized cells were stained with a PE-labeled Ab to Erk1 and Erk2 (BD), according to the manufacturer's instructions. Cells were fixed with 2% paraformaldehyde, and all data were collected and analyzed as done for standard flow cytometry.

### Immunoblotting and phosphotyrosine analysis

5×10^6^ splenic BALB/c B cells were isolated by negative depletion of CD43-expressing cells with anti-mouse CD43 MACS beads (Miltenyi Biotech) to >95% purity, as assessed by flow cytometry. Purified B cells were equilibrated to 37°C in a humidified incubator and stimulated with gp41 MPER tetramers or F(ab)'2 anti-IgM (Jackson ImmunoResearch) in 5% FBS-containing DMEM media for various times. Whole cell extracts were made by lysing cell pellets in 1% NP-40 lysis buffer supplemented with PhosSTOP and complete, mini EDTA-free protease inhibitor tablets (Roche) and insoluble cellular debris was removed by centrifugation. Extracts were fractionated by reducing SDS PAGE on Novex Bis-Tris Mini gels (Invitrogen) and transferred to Invitrolon PVDF membranes (Invitrogen) using the Xcell II blot module. Primary Abs used were anti-phosphotyrosine clone 4G10 (Upstate) and anti-GAPDH (Millipore). Secondary Abs used were goat-anti-mouse IgG1 or IgG2a, both coupled to HRP (Jackson ImmunoResearch). Blots were developed using the WesternLightning ECL system (Perkin Elmer).

### Production of IgM fragments and Surface Plasmon Resonance (SPR)

2F5 was purchased from Polymun and the CD4-inducible anti-HIV-1 mAb 17b was provided by Dr. J. Robinson. Purified mouse pentameric IgM^a^, BD clone G155-228 and IgM^b^, BD clone C48-6 (both derived from J558 V_H_-expressing, TNP-KLH-specific mAb cell lines) were fragmented into monomeric or F(ab)_2_ fragments and purified using the IgM fragmentation kit (Pierce), according to the manufacturer's instructions. Purity of fragments was confirmed by running fragments on non-reducing gels and staining with Coomassie Blue. All SPR measurements of IgM fragment interactions with the gp41 MPER epitope were conducted on a BIAcore 3000 instrument, and data analyses, including affinity measurements, were performed using the BIAevaluation 4.1 software (BIAcore), as previously described [31].

## Supporting Information

Figure S1 Cross comparison of HIV-1 Env-specific B cell tetramer binding to mAb-coated beads and mAb cell lines. A) Beads coated with human mAbs 7B9 and F39F (specific to the HIV-1 Env gp120 V3 loop, previously described in [8], [77]), the human mAb 7B2 (specific to the HIV-1 Env gp41 immunodominant (ID) domain; kindly provided by James Robinson, Tulane University), the human mAb A32 (specific to the the HIV-1 Env CD4 binding site; previously described in [78]), and the human mAb 13H11 (a non-neutralizing mAb specific to the HIV-1 Env gp41 MPER epitope; previously described in [40]), respectively were either left unstained (filled histograms) or stained with various HIV-1 Env-specific B cell tetramers (open histograms). Note that epitope-specific mAbs only bound tetramers bearing the respective cognate epitope and did bind tetramers with their respective epitopes scrambled. P3X63/Ag8 (previously described in [77]) is a mAb lacking HIV-1 epitope specificity and was used as additional negative control for tetramer binding. B) mAb hybridoma cell lines P3X63/Ag8, F39F, 7B2, and 13H11 were either left unstained (filled histograms) or reacted with various HIV-1 Env-specific B cell tetramers (open histograms). Surface immunoglobulin expression on cell lines was demonstrated by staining with anti-mouse-IgG (P3X63/Ag8 and 13H11) or anti-human-IgG (F39F and 7B2) reagents.
(1.62 MB EPS)

Figure S2 Surface plasmon resonance analysis of C57BL/6 and BALB/c naïve serum reactivity to the gp41 MPER 2F5 epitope. 1∶5 dilutions of serum from unimmunized 12 wk-old BALB/c and C57BL/6 mice or 10 micrograms of purified 2F5 mAb (used as a positive control) were captured over biotinylated gp41 MPER peptide, anchored to an L1 sensor chip, as described in Materials and Methods. Results are representative of two independent experiments.
(0.26 MB EPS)

Figure S3 Surface plasmon resonance analysis comparing IgMa and IgMb binding to various gp41 MPER peptides. 10 micrograms of purified monomeric or F(ab)2 fragments derived from TNP-KLH-specific IgMa and IgMb mAbs were injected over the following biotinylated peptides: gp41 MPER (aka Sp62, containing the 20 aa “optimal/higher affinity” gp41 MPER 2F5 epitope), scrambled gp41 MPER, gp41 MPER (gp120 Env642-678; aka DP178Q16L, containing a “lower-affinity” gp41 MPER 2F5 epitope, comprised of 16 additional N-terminal residues of the gp41 MPER HR-2 region), and gp41 HR1, a peptide spanning a region of the gp41 MPER outside the 2F5 epitope. Results are representative of two independent experiments.
(0.33 MB EPS)

Figure S4 Surface plasmon resonance analysis of IgMa and IgMb F(ab)2 fragment binding to TNP-BSA. 10 micrograms of purified F(ab)2 fragments derived from the TNP-KLH-specific IgMa and IgMb mAbs (BD clones G155-228 and C48-6, respectively) were injected over a sensor chip immobilized with a 2,4,6-Trinitrophenyl hapten-Bovine Serum Albumin conjugate (TNP-BSA, conjugation ratio 2 (T-5050-10), Biosearch Technologies Inc.). Results were subtracted from a sensor chip coated with BSA alone, and mAb 13H11 (specific for gp41 MPER, but non-reactive with TNP) was used as a negative control. Results are representative of two independent experiments.
(0.27 MB EPS)

Table S1 (0.64 MB EPS)

Table S2 (0.52 MB EPS)

Table S3 (0.29 MB EPS)

Table S4 (0.48 MB EPS)
